# Genetic Ancestry Inference and Its Application for the Genetic Mapping of Human Diseases

**DOI:** 10.3390/ijms22136962

**Published:** 2021-06-28

**Authors:** Eva Suarez-Pajes, Ana Díaz-de Usera, Itahisa Marcelino-Rodríguez, Beatriz Guillen-Guio, Carlos Flores

**Affiliations:** 1 Research Unit, Hospital Universitario Nuestra Señora de Candelaria, 38010 Santa Cruz de Tenerife, Spain; eva.sp.95@gmail.com (E.S.-P.); itahisa@gmail.com (I.M.-R.); bguillenguio@gmail.com (B.G.-G.); 2 Genomics Division, Instituto Tecnológico y de Energías Renovables (ITER), 38600 Santa Cruz de Tenerife, Spain; adiaz@iter.es; 3 CIBER de Enfermedades Respiratorias, Instituto de Salud Carlos III, 28029 Madrid, Spain

**Keywords:** admixture mapping, genetic ancestry, ancestry informative markers, next-generation sequencing

## Abstract

Admixed populations arise when two or more ancestral populations interbreed. As a result of this admixture, the genome of admixed populations is defined by tracts of variable size inherited from these parental groups and has particular genetic features that provide valuable information about their demographic history. Diverse methods can be used to derive the ancestry apportionment of admixed individuals, and such inferences can be leveraged for the discovery of genetic loci associated with diseases and traits, therefore having important biomedical implications. In this review article, we summarize the most common methods of global and local genetic ancestry estimation and discuss the use of admixture mapping studies in human diseases.

## 1. Genetic Admixture

Admixed populations are the result of gene flow between reproductively isolated groups, owing to events that have occurred throughout human history, including migratory events, the discovery of new territories, or the slave trade. As a result of the intermixture and recombination, over time, the genomes of individuals in the hybrid population will contain a mosaic of ancestries from different population sources in their chromosomes. The length of the chromosome segments inherited from the different ancestral populations will be proportional to the time elapsed since the admixture event. These tracts shorten over the generations by the meiotic recombination process, so that the most recently admixed populations, such as the Canary Islanders in Spain or the Latino populations, would retain longer ancestral tracts, while the populations that mixed more distantly in time, such as the Uyghur in China, would harbor shorter ancestry segments in their chromosomes [1,2].

As such, the admixture proportions and the elapsed time since the admixture event can be inferred based on linkage disequilibrium (LD) [3,4]. When two distant populations interbreed, the admixture linkage disequilibrium (ALD) can be generated among loci with different allelic frequencies in the ancestral populations, leading to a linkage between markers that were previously unlinked. During the first generations since the admixture, ALD is expected to experience a rapid decay between distant loci, while it would be maintained between closer positions and can be detected after generations [5]. Additionally, the ALD dynamics of decay are also influenced by the admixture model. For example, a greater drop of ALD and a faster length decrease in the ancestral chromosomal segments are expected for those populations that have been formed by a single mixing event, compared with admixtures maintained throughout generations [1,6].

Several studies support that genetic ancestry and admixture can partially explain the differences in the prevalence of complex diseases and treatment responses between population groups, due to the unequal distribution of allelic frequencies of the underlying causal variants across populations. As such, and given that the prevalence of many traits and diseases differs between populations, the analysis of genetic ancestry differences between affected and nonaffected subjects, as well as the link of these differences with the pathogenesis, and with evolutionary, environmental, or behavioral factors, plays an important role in biomedical research. Some examples are the high prevalence of multiple sclerosis among Europeans [7], of diabetes in North American and Caribbean regions [8], and of hypertension in African and Asian populations [9].

Many studies also support the existence of genetic variants affecting drug metabolism, transport, and toxicity, which vary widely among populations and ethnic groups [10,11]. In this sense, the response to the anticoagulant warfarin has been studied in different admixed populations in order to explain how genetic ancestries could contribute to the interindividual response to the treatment [12]. In addition, similar studies have developed algorithms to predict warfarin doses and improve the treatment in Hispanic-Caribbeans [13,14]. Furthermore, genetic variants of Cytochrome P450 Family 2 Subfamily C Member 19 (*CYP2C19*) and Paraoxonase 1 (*PON1*) genes, which are highly structured by ancestry, modify the response of the antiplatelet agent clopidogrel in Puerto Ricans [15]. Additionally, among many others, the relationship between genetic ancestry and the response to bronchodilators in patients with asthma [16] or to treatments for acute lymphoblastic leukemia has been investigated [17].

Given the importance of genetic ancestry in medicine, here, we provide an updated review of the most common methods for global and local genetic ancestry estimation, and of their use in admixture mapping approaches, highlighting a few key findings from the recent literature. We also discuss the potential of Next Generation Sequencing (NGS) data for ancestry estimation.

## 2. Estimation of Genetic Ancestry: Global and Local Ancestry

**Figure 1:**
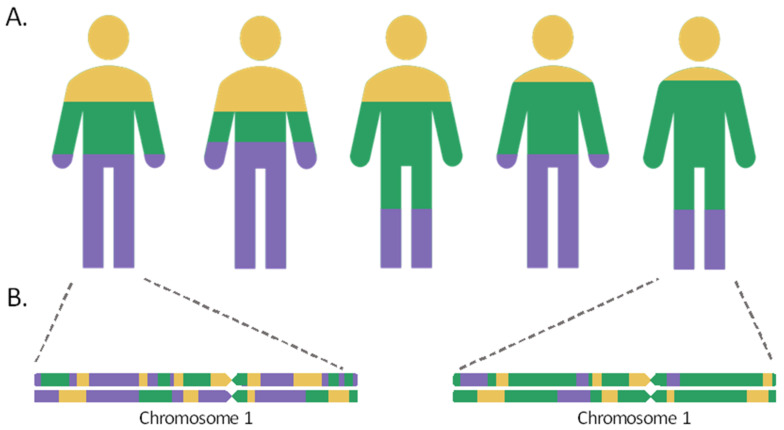
Global (**A**) and local (**B**) genetic ancestries in a recently admixed population with three ancestral populations. The proportion of each of the ancestral populations is represented by the colors yellow, blue, and purple.

Global ancestry (GA) is the fraction of genomic ancestry from each admixed individual that can be ascribed to each of the ancestral populations contributing to the recently admixed population (Figure 1A). The estimate of GA can be obtained using different approaches. Some of the most popular methods are based on probabilistic models using genotype data, assuming that populations are in Hardy–Weinberg equilibrium and considering complete linkage equilibrium for all loci considered for the estimation, such as STRUCTURE [18,19] and ADMIXTURE [20,21]. Alternative approaches that allow the estimation of the ancestry proportions are based on principal component decompositions, such as ipPCA [22], and on the study of LD decay curves, such as ALDER [3].

Local ancestry (LA) is a term commonly used to refer to the ancestry in each of the chromosome blocks, also known as ancestral tracks, in recently admixed individuals (Figure 1B). For this, the number of copies derived of each ancestral population, in each genomic position, could be inferred per individual (from zero to two copies). Thus, GA can also be obtained by summarizing LA across the individual genomes. Multiple estimators have been developed to infer LA (Table 1). Briefly, the choice of the most appropriate approach will depend on the number/density of available markers as well as on the evolutionary history of the admixed population under study. Some models are based on haplotype data and require specific reference panels that may not be available for all populations [23,24,25,26]. Furthermore, LA inference is complicated in scenarios of admixture between populations with a limited genetic divergence, or of an old admixture event.

Some of the genetic characteristics of recently admixed populations described above (chromosome blocks and ALD distribution) allow the estimation of ancestry with a relatively small number of genetic markers. These markers are usually single nucleotide polymorphisms (SNPs) with a different distribution between populations, known as ancestry informative markers (AIMs). Their number will also depend on the assessed population, the ancestral groups, and the time since the admixture event [27]. In order to identify those markers that are useful and informative of ancestry, multiple measurements of population differentiation have been proposed [28,29]. Additionally, the widespread use of SNP arrays and NGS technologies with information from hundreds of thousands genetic markers has facilitated LA inferences and the development of AIMs panels for particular admixed populations [30,31,32].

**Table 1:** Most common methods to estimate local genetic ancestry.

SOFTWARE	Algorithm	Background LD	Phasing Requirement	Genetic Map	Physical Map	Number of Ancestral Populations	Reference
CHROMOPAINTER	HMM	Yes	Phased	Optional	No	≥2	[33]
EILA	k-means	No	Unphased	No	Yes	2 or 3	[34]
ELAI	Two layers HMM	Yes	Phased/Unphased ^a^	No	No	≥2	[35]
HAPMIX	HMM	Yes	Phased /Unphased ^b^	Yes	No	2	[36]
LAMP-LD	HMM	Yes	Phased/Unphased ^b^	No	Yes	2, 3 or 5	[37]
Loter	Single layer HMM	No	Phased	No	No	≥2	[23]
PCAdmix	HMM and local PCA	No	Phased	Optional	Optional	≥2	[25]
RFMIX	CRF	No	Phased	Yes	No	≥2	[24]
SABER +	HMM	Yes	Phased	No	No	2–4	[38,39]
SEQMIX	HMM	No	Unphased	Yes	No	2	[40]
SupportMix	SVM	No	Phased	Yes	No	≥2	[26]

^a^ Phased and unphased data are allowed for ancestral and admixed populations. ^b^ Phased data are needed for the ancestral populations and unphased data for the admixed population. CRF (Conditional Random Field), HMM (Hidden Markov Model), LD (linkage disequilibrium), PCA (Principal Component Analysis), SVM (Support Vector Machines).

The use of genotyping microarrays has also led to the development of improved methods to infer LA, such as LAMP-LD [37], RFMix [24], and HAPMIX [36], among others (Table 1). Compared to the previous methods that were designed to deal with AIMs, these other algorithms rely on denser sets of genetic markers (retaining LD) that allow one to obtain a higher resolution in estimating LA, most of them based on hidden Markov models [35,37]. Additionally, the development of machine learning methods has allowed the use of algorithms such as random forests [24], which have been suggested to provide more accurate estimates [41].

In order to identify the optimal approach for each scenario, benchmarking the different algorithms and reference panels is necessary. Previous reviews have compared the characteristics and effectiveness of local ancestry estimators [42,43,44,45,46], suggesting a few main aspects to consider: (1) the prior requirements of each estimator, and (2) the inherent features of the target population itself. Table 1 shows the main characteristics of the most common methods to estimate local ancestry.

Regarding the necessary requirements for the use of each estimator, it must be considered, for example, whether a phasing step is needed prior to ancestry estimation. This step is crucial for an accurate estimate of ancestry and is closely linked to the density of available markers [47]. Therefore, the use of an algorithm not requiring a prior phasing step (e.g., EILA or SEQMIX) may be less biased. On the other hand, certain tools need specific marker information, such as their intermarker distance (physical maps) and/or the recombination rate (genetic maps). Additionally, other options such as HAPMIX [36], ELAI [35], RFMIX [24], and SUPPORTMIX [26] require imposing a number of generations since the occurrence of the last admixing event. Therefore, uncertainty or the lack of accurate population information can lead to biased estimates.

Moreover, the inherent characteristics of the population under study can also influence software selection. For example, although RFMIX [24] or LAMP-LD [37] have reported accurate local ancestry estimates, their effectiveness is reduced when increasing the number of generations since the admixing event. In the case of ancient admixtures, Loter [23] and ELAI would allow one to obtain better estimates [23,43]. On the other hand, the number of ancestral populations of the target population is also an important factor to be considered in the selection of the estimator (Table 1), as well as the magnitude of the differentiation among the parental populations. For example, ELAI has been shown to better differentiate North African and sub-Saharan ancestry components than has LAMP-LD [2].

Finally, another important aspect that must be considered is the computational requirements of each software, such as the execution time or memory used, which is strongly conditioned by the sample size of the admixed sample under study [43,44]. In this sense, Loter [23] or RFMIX [24] offer the possibility of reducing the computation time via parallel implementation, splitting the process into multiple threads.

## 3. Admixture Mapping Studies

### 3.1. Definition

**Figure 2:**
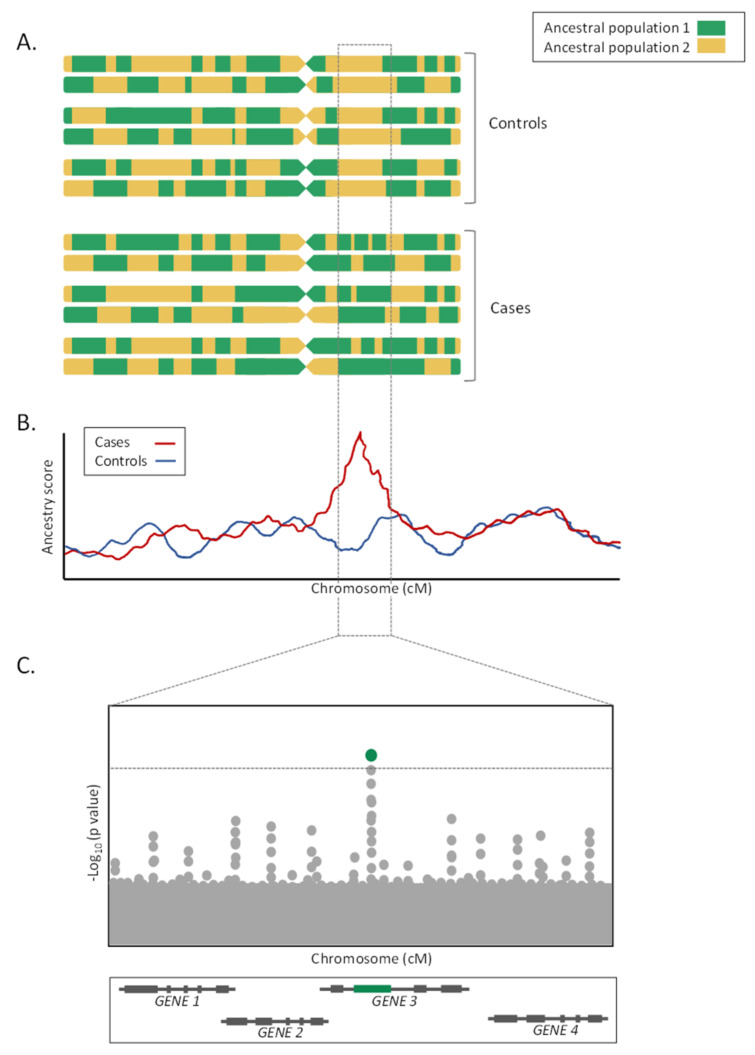
Scheme of an admixture mapping study. (**A**) LA estimates in cases and controls individuals from a recently admixed population. (**B**) Comparison of local ancestry scores of all chromosomal regions between cases and controls. (**C**) Fine mapping study on genomic regions where genetic ancestry is associated with a trait.

The distribution of allelic frequencies in recently admixed populations is closely related to those frequencies found in their ancestral populations [48,49]. When these ancestral populations have marked differences in the susceptibility to a disease, admixture mapping studies, also known as mapping by admixture linkage disequilibrium (MALD) studies, can be performed to reveal genetic loci harboring variants underlying such differences between population groups [50]. Admixture mapping studies aim to correlate LA with a trait of interest in recently admixed populations in which ALD is still detectable, under the hypothesis that variants associated with increased disease risk will be found in chromosomal fragments inherited from one of the parental populations [51,52]. Thus, an increment (or decrease) in the proportion of the ancestry associated with the trait of interest will be expected in these chromosomal regions (Figure 2, Table 2).

Admixture mapping studies can be performed using case-only or case-control approaches. The case-only design is based on the comparison between observed and average expected ancestry to detect loci enriched in one of the ancestries, while the case-control approach consists of comparing LA pointwise between cases and controls [53,54]. Although case-only studies have shown a greater higher statistical power, case-control analyses are less biased when it comes to verifying that the changes in ancestry are due to the association with the trait of interest and not to other confounding factors [53,54]. Different admixture mapping tools have been developed to perform LA association analyses, both for case-only and for case-control approaches. Classically, the most widely used tools to perform an admixture mapping are ADMIXMAP [55], ANCESTRYMAP [56], or MALDsoft [54]. Additionally, LA estimations could be used as a proxy of the genotype variable within logistic regression models for association study testing on the admixed individuals [57,58]. In the event that the study population presents kinship relationships, it is necessary to use specific methods to correct the population structure, such as PC-Air [59] or EMMAX [60].

### 3.2. Advantages and Disadvantages of Admixture Mapping Studies

Since 2005, genome-wide association studies (GWAS) have allowed the identification of thousands of genetic loci associated with many traits, including complex diseases [61]. However, as of June 2020, nearly 90% of GWAS reported in the GWAS Catalog were obtained from Europeans [62]. Thus, a significant part of the genetic variability exclusive to or better represented in other ethnic groups remains largely unexplored, which has obvious consequences for the generalized implementation of precision medicine. Assuming that the effect of the risk variants can vary depending on the surrounding genetic variation, some results could be affected by the hidden stratification caused by the LA or if the direction of the effect is opposite in the distinct ancestral populations [58,63]. In this sense, admixture mapping studies overcome these limitations, offering a more efficient alternative to contribute to the disentangling of the genetic architecture of diseases and traits in recently admixed populations. As a major advantage, given that LA tracks are usually large, often measured in the megabase-scale, the significance penalty of these studies is much lower than for GWAS, therefore increasing the statistical power for a given sample size [48]. Furthermore, these studies are less affected by allelic heterogeneity than GWAS, because they are based on LA and not on SNP alleles directly.

On the contrary, admixture mapping studies lose efficiency when allele frequencies are similarly distributed among ancestral populations and when the LD in the parental populations is unknown [64,65]. Furthermore, for the LA estimations to be accurate, the choice of the LA estimator, as well as the number and density of genotyped markers, is critical [65]. Additionally, given the megabase size of the loci detected by the admixture mapping approaches, fine mapping studies or candidate gene association studies focused on the prioritized genomic regions must follow for the study to be fully completed (Figure 2C). Finally, as in the GWAS, admixture mapping approaches only allow the detection of the genetic risks associated with the trait of interest, and do not consider the environmental, cultural, or socioeconomic factors [64].

### 3.3. Applications of Admixture Mapping Studies in Biomedical Research

Based on admixed populations, several studies have implemented admixture mapping approaches to reveal novel risk factors associated with complex diseases, including cancer, hypertension, and autoimmune, respiratory, and infectious diseases. Primarily, these studies have been widely applied in African Americans and Hispanic/Latino populations. The genetic contribution of each of the parental populations has been estimated for African Americans from four different states, providing an estimate of average ancestry corresponding to 76.4% African, 20.9% European, and 2.7% Native American [66]. A similar approach was carried out in the Hispanic population of Southern Colorado, for whom the composition was reported to correspond to 62.7% European, 34.1% Native American, and 3.2% African [67]. However, these estimates differ widely between the states [68]. In this sense, recent studies in African American populations have found genomic regions with increased proportions of African ancestry associated with risk of prostate and breast cancer [69,70,71]. Additionally, Schwartz and colleagues conducted admixture mapping studies using both case-only and case-control approaches in 1,812 African Americans, and revealed two loci linked to lung cancer susceptibility, one of them at 1q42 with an excess of European ancestry, and another at 3q25 enriched in African ancestry [72]. Furthermore, Yang and colleagues reported genomic loci enriched in Native American ancestry that could influence relapse and a poorer prognosis for lymphoblastic leukemia in a heterogeneous cohort of self-reported ancestries [17].

Zhu and colleagues performed an admixture mapping in African Americans, identifying an excess of African ancestry at 6p24 and 21p21 associated with hypertension [73]. Gignoux and colleagues detected a genomic region in 18q12 in the Latino population, where an increase in Native American ancestry was associated with a greater asthma risk, while European ancestry was associated with asthma protection [74]. Furthermore, an increased risk of multiple sclerosis was linked to European genetic risk factors in African American and Hispanic individuals [75]. Admixture mapping studies have also been used to explain why some populations are more susceptible to suffer infections caused by microbes. For instance, admixture mapping studies allowed explanations for the susceptibility to tuberculosis caused by *Mycobacterium tuberculosis* in the South African Colored population [76] and the incidence of *Staphylococcus aureus* in African Americans [77], allowing the detection of genomic regions that could be involved in the pathogenesis. Furthermore, these approaches have also been applied to investigate the treatment response to identify the genetic differences that modify the response to drugs. Recent studies suggest that genetic variants related to drug metabolism, transport, and toxicity may vary between ethnic groups [10,11]. Therefore, admixture mapping approaches are necessary to study the implication of the genetic ancestry in the response to a treatment. In this sense, Spear and colleagues found an African region at 8p11 with a suggestive association to bronchodilator response in African Americans patients with asthma [78].

Additionally, although less frequently, admixture mapping studies have also been performed in admixed populations other than Latinos or African Americans. For instance, Sun and colleagues recently performed an admixture mapping approach in the Hawaiian population in order to determine the relation of the genetic admixture with different cardiovascular traits. Interestingly, this admixed population (78% Native Hawaiian, 11.5% European, and 7.8% Asian [79]) has a high burden of cardiovascular diseases and diabetes [80,81]. Despite that, it has been a poorly studied minority. The study revealed an excess of native Hawaiian ancestry on chromosome 6, and further single variant association tests focused on this region identified a variant in the 5’UTR region of the eyes shut homolog (*EYS*) gene significantly associated with type 2 diabetes [82]. Moreover, this strategy has been applied to recently admixed western European populations, specifically in the Spanish population of the Canary Islands in the Macaronesia, where the use of SNP arrays and whole-genome sequencing revealed that average proportions of ancestry correspond to 75% European, 22% North African, and 3% South Saharan African [2]. There is only one admixture mapping study in the European population published to date. This was performed by Guillen-Guio and colleagues, who performed an admixture mapping study of asthma in the Canary Island population [83]. This study allowed the detection of a novel locus within chromosome 16 (16q23.3) enriched in North African ancestry that was associated with asthma risk. Subsequent whole-exome sequencing analyses revealed that the phospholipase C gamma 2 (*PLCG2*) gene, located in that region, was enriched of deleterious variants among asthma cases [83]. Likewise, other populations that could be excellent targets for admixture mapping studies are the Cape Verde population, also from the Macaronesia, since they are a result of recent gene flow between Africans and Europeans [84], and the Uyghur population, which can be modelled by a two-way admixture between Europeans and East Asians [31]. Genetic ancestry studies could assist in unraveling the genetics underlying prevalent diseases in these populations, helping to raise the representation of diversity in the genetic architecture of diseases, and thus result in a better transference of knowledge to personalized medicine.

## 4. NGS and Genetic Ancestry Estimation

The technological development of NGS over the years, together with the reduction in the sequencing costs, offers a great opportunity for genetic ancestry studies to develop further. Among the major advantages of this technology compared to microarrays (Table 3) is its high-throughput capacity, resulting in thousands of DNA fragments being sequenced simultaneously, offering the possibility of covering a larger fraction of the genome. Therefore, the use of NGS, especially of whole-genome sequencing (WGS), allows an increase in the number of markers tested to infer LA, and the possibility to find optimal ancestry-specific genetic markers. This permits one to obtain reference panels of ancestral populations and then design panels of more restricted AIMs, as done by Li-Ju Wang and colleagues, who proposed a specific panel of AIMs to infer three-way genetic admixture (European, East Asian, and African) by using whole-exome sequencing (WES) data [85]. Furthermore, the NGS technology allows the detection of information from the entire spectrum of allelic frequencies, from common variants to low-frequency and rare variants, which, by definition, are expected to be more structured among populations compared to the common variation that is typically covered by most SNP genotyping microarrays. This leads to better detection of population-specific variants and, therefore, improved LA estimation [86]. Additionally, another advantage offered by the NGS is that detected SNPs are not affected by ascertainment bias, which is induced by an incorrect or nonrepresentative selection of markers. In this sense, Maróti and colleagues assessed the used of WGS, WES, and SNP genotyping microarray data in population genetic analyses [87]. Their results suggested that SNP genotyping data may be more prone to biasing the results, as they are related to significantly higher cross-validation error values and an overestimation of the admixture proportions than are WES or WGS data. Accordingly, Lachance and Tishkoff suggested that the use of biased markers from genotyping arrays may misestimate LD and overestimate population differences [88]. Since these aspects are important for LA inference and, consequently, for the proper performance of an admixture mapping study, we anticipate that the use of NGS will lead to more accurate estimates.

However, although the application of NGS implies important advances, its use to estimate LA still has important limitations (Table 3). First, the large amount of data generated by NGS approaches implies a large computational requirement and high economic costs. Second, most existing algorithms have been designed for SNP genotyping microarray data (e.g., LAMP-LD limits the use to datasets of markers up to 500,000 SNPs [37]). Additionally, the analysis of WGS data using methods not optimized for NGS can lead to decreased accuracy of the LA estimation [86]. To cope with this limitation, specific NGS software is being developed for ancestry estimation, mainly aimed at WES analysis [40,86]. Third, the LA estimation is conditioned by the depth of coverage of the sequencing data, as a low depth of coverage increases the likelihood of introducing false genotype calls that could lead to biases in the estimation of the ancestry proportions. Different tools have recently been developed to estimate LA addressing this problem. For example, SEQMIX, which has been optimized to use low depth of coverage data from WES or targeted sequencing, allows one to obtain accurate estimations by combining data from off-target and on-target reads [40]. Lanc-CSV uses continent-specific variants (CSV) (i.e., variants that are private to one of the continental populations) and preprocessing steps to guarantee a minimal depth of coverage in CSV for them to be considered further downstream for LA estimation [86]. Finally, considering that WES covers only 1–2% of the genome, the inference of ancestry blocks using WES data is reduced to this portion of the genome. In this sense, new methods to infer the variation of uncovered regions have been developed and could be applied to improve LA inference [89].

Although the blooming use of NGS represents a great opportunity for population studies improvement, only two NGS-based admixture mapping studies have been published to date [90,91]. Liu and colleagues performed an admixture mapping study of blood pressure phenotypes using WES data from African American individuals to under-stand their higher prevalence of hypertension, revealing four regions enriched in African ancestry linked to diastolic blood pressure, two of them also overlapping with regions that were significantly associated with mean arterial pressure [90]. Additionally, Lin and colleagues accomplished a multi-ethnic study using WGS data to study glomerular phenotypes. Although no significant associations were detected in the admixture mapping study, using WGS data allowed them to identify three rare variants associated with estimated glomerular filtration rate [91]. Therefore, although the ancestry inference using sequencing data continues to be a challenge, the results of these two studies support the huge potential for the use of NGS in admixture mapping approaches.

## 5. Concluding Remarks

Genetic ancestry studies and admixture mapping approaches have expanded genetic knowledge in biomedical research, revealing new loci associated with traits and diseases that could not have been detected by conventional association studies. Despite this, the available genomic resources need to be improved to obtain more accurate ancestry inferences. For instance, WGS data for African or Middle Eastern populations are still limited. Interestingly, the genetic features of Arab populations could be a valuable opportunity, especially in the study of recessive traits given the high rate of consanguinity that could be overlooked in other population groups [62]. In this context, additional deep characterizations of the genetic variation on these populations are needed to further improve estimations and reduce biases or false associations caused by the lack of proper reference datasets. Therefore, initiatives such as The Human Heredity and Health in Africa (H3Africa) consortium have been developed in order to promote genetic and environmental base studies of human diseases in Africans and their clinical application [92,93]. On the other hand, the National Arab Genome Project in the United Arab Emirates (UAE) aims to achieve a greater representation of Arab genomes through NGS technologies [94].

Consequently, a more equitable representation of the ancestral groups in association studies will improve the development of personalized medicine. The unequal proportion in European-based GWAS is known to generate a bias in the findings that could lead to misinterpretations and hamper the risk prediction, prognosis, or response to drugs in under-represented populations [95]. In this regard, Manrai and colleagues detected that patients of African ancestry with hypertrophic cardiomyopathy received reports with misclassified risk variants [96]. This highlights the need to expand the catalog of genetic variation across diverse populations, as well as to promote studies in non-European populations in order to improve the prediction and treatment of diseases for individuals, irrespective of their ancestry.

There are other approaches currently used in biomedical research that would benefit from taking genetic ancestry into account. Gene expression studies, which seek to explain how the expression of certain genes influences the development or severity of a trait, are an example. Based on the evidence that gene expression levels also differ between populations, in recent years, there has been a need to incorporate ancestry in expression studies and generate ancestry-dependent transcriptomic profiles [97,98,99]. Additionally, polygenic risk score (PRS) modeling, which uses a composite of the individual risk variant effects, estimated from association studies, to predict the overall genetic risk of developing a particular trait, have relied primarily on European GWAS data. Therefore, the transferability of the PRS scores to other population groups is limited and, as a consequence, a worse performance has been described in African populations [100]. Therefore, there is an urgent need to include other admixed populations and to consider genetic ancestry in these models, to optimize the PRS, and improve the efficiency of this approach [101,102]. Finally, recent studies show that rare or low-frequency variants are more likely to have a larger effect on complex traits [103,104]. Most studies that have focused on rare variants have been designed for genetically homogeneous populations and do not consider the effect of local genetic ancestry. Recently, Qin and colleagues have developed an approach to identify chromosomal blocks that harbor rare variants using the local ancestry, which has shown more powerful results than other methods for the case of admixed populations [105]. In this sense, increasing the knowledge in this field will allow for a better understanding of how these uncommon variants influence diseases and traits.

In summary, promoting genetic studies in admixed populations, and the use of admixture mapping studies, combined with the alternative approaches described, promise the identification of novel disease associations and a better understanding of complex trait genetics. Eventually, these results will translate into a more equitable representation of the catalogs of genetic variation across populations.

## Figures and Tables

**Table 2:** Definition of the main concepts.

Concept	Definition
Ancestry informative marker (AIM)	Genetic variants, usually SNPs, that show large frequency differences between the parental populations and that are, thus, highly informative for ancestry estimation in admixed populations.
Admixture model	A simple model to describe how gene flow between ancestral populations could have occurred. Admixed populations can be the result of a mixture between individuals from two or more populations and that can be maintained in various generations (gradual admixture) or be a result of a single event (hybrid isolation).
Ancestry estimation	In admixed populations, this allows the determination of the proportion of each of the ancestries for a given admixture model.
Global ancestry (GA)	Estimated ancestry proportion with which each parental population contributes on average to the genome of an admixed individual for a given admixture model.
Local ancestry (LA)	Estimated ancestry proportion with which each parental population contributes to each locus of the genome of an admixed individual for a given admixture model.
Admixture mapping	Method that allows detecting if the genetic ancestry of a particular section of the genome in a mixed population tends to be inherited with a particular trait.

**Table 3:** Advantages and disadvantages of using NGS for LA estimation.

Advantages	Disadvantages
Larger fraction of the genome covered. Detection of low-frequency and population-specific variants. More accurate LA estimate. SNPs not affected by ascertainment bias.	Lack of specific algorithms and software. Accuracy depends on sequencing coverage. WES covers a small portion of the genome. Higher computational and economic costs.

LA (Local ancestry), WES (Whole-Exome Sequence), SNP (Single Nucleotide Polymorphism).

## Data Availability

Not applicable.
